# Antitumor potential of the myotoxin BthTX-I from *Bothrops jararacussu* snake venom: evaluation of cell cycle alterations and death mechanisms induced in tumor cell lines

**DOI:** 10.1186/s40409-015-0044-5

**Published:** 2015-11-03

**Authors:** Cássio Prinholato da Silva, Tássia R. Costa, Raquel M. Alves Paiva, Adélia C. O. Cintra, Danilo L. Menaldo, Lusânia M. Greggi Antunes, Suely V. Sampaio

**Affiliations:** Department of Clinical Analyses, Toxicology and Food Sciences, School of Pharmaceutical Sciences of Ribeirão Preto, University of São Paulo (USP), Avenida do Café, s/n, Ribeirão Preto, SP CEP 14040-903 Brazil

**Keywords:** *Bothrops jararacussu*, BthTX-I, Antitumor potential, Apoptosis, Cell cycle alterations

## Abstract

**Background:**

Phospholipases A_2_ (PLA_2_s) are abundant components of snake venoms that have been extensively studied due to their pharmacological and pathophysiological effects on living organisms. This study aimed to assess the antitumor potential of BthTX-I, a basic myotoxic PLA_2_ isolated from *Bothrops jararacussu* venom, by evaluating *in vitro* processes of cytotoxicity, modulation of the cell cycle and induction of apoptosis in human (HL-60 and HepG2) and murine (PC-12 and B16F10) tumor cell lines.

**Methods:**

The cytotoxic effects of BthTX-I were evaluated on the tumor cell lines HL-60 (promyelocytic leukemia), HepG2 (human hepatocellular carcinoma), PC-12 (murine pheochromocytoma) and B16F10 (murine melanoma) using the MTT method. Flow cytometry technique was used for the analysis of cell cycle alterations and death mechanisms (apoptosis and/or necrosis) induced in tumor cells after treatment with BthTX-I.

**Results:**

It was observed that BthTX-I was cytotoxic to all evaluated tumor cell lines, reducing their viability in 40 to 50 %. The myotoxin showed modulating effects on the cell cycle of PC-12 and B16F10 cells, promoting delay in the G0/G1 phase. Additionally, flow cytometry analysis indicated cell death mainly by apoptosis. B16F10 was more susceptible to the effects of BthTX-I, with ~40 % of the cells analyzed in apoptosis, followed by HepG2 (~35 %), PC-12 (~25 %) and HL-60 (~4 %).

**Conclusions:**

These results suggest that BthTX-I presents antitumor properties that may be useful for developing new therapeutic strategies against cancer.

## Background

According to the World Health Organization, cancer is one of the leading causes of morbidity and mortality worldwide. Cancer is a generic term for a large group of diseases that may affect any part of the body [1]. These diseases are characterized by uncontrolled growth and spread of abnormal cells, and may be caused by external factors (smoking, infectious organisms, radiation and chemical products) and internal factors (inherited or metabolic mutations, hormones and immune conditions) [2].

A correct diagnosis of cancer is essential for appropriate and effective treatment, because each cancer type requires specific treatments that may include surgery, radiotherapy and/or chemotherapy. Nonetheless, chemotherapy is flawed especially considering that no chemotherapeutic agent available acts exclusively on tumor cells, affecting also many types of normal body cells and leading to several undesirable effects [2, 3].

Currently there is great medical and scientific interest in the investigation of natural products for therapeutic purposes, in search of new molecules that could be used as antitumor agents with fewer side effects than the usual chemotherapy, or serve as molecular models for the development of more effective drugs against malignant tumors [4, 5].

In recent years, studies on snake venoms and their components have demonstrated the antitumor effects of different toxins [6–8]. The focus of these researches has been the understanding of the mechanisms of action of toxins on different types of tumors. It is known that the cytotoxicity induced by animal venoms is mainly related to changes in the cellular metabolism of cells, especially tumorous ones, however, the mechanisms of action of these toxins are still to be clarified, which makes them target of many studies with interest in their therapeutic potential [8].

Some phospholipases A_2_ (PLA_2_s) have been described to possess antitumor and antiangiogenic properties [9–11]. PLA_2_s are enzymes of great scientific interest due to their involvement in several human inflammatory diseases and in envenomations by snakes and bees. In addition, PLA_2_s present important effects on lipid membranes and are related to the formation of various substances involved in numerous biological activities, such as prostaglandins, prostacyclins, thromboxanes and leukotrienes [12, 13].

Myotoxins are PLA_2_ homologues that present a substitution of the aspartic acid residue at position 49 (Asp49) by a lysine (Lys49), with changes in their calcium binding site. These variations are related to the low or no catalytic activity presented by these molecules [14]. Nevertheless, myotoxic PLA_2_s have diverse biological effects, such as myotoxicity, edema, anticoagulation, neurotoxicity, indirect hemolysis, cytotoxicity, and also antibacterial, antiviral and antiparasitic activities [15].

Bothropstoxin-I (BthTX-I) is a single chain protein of 13.7 kDa and pI 8.2, with 121 amino acid residues and 7 disulfide bonds, isolated from *Bothrops jararacussu* snake venom [16]. This basic myotoxin showed action on the gastrocnemius muscle of mice and cytotoxicity in murine muscle cells (C2C12) and also had its cytotoxic effects evaluated both *in vitro* and *in vivo* on different tumor cell lines such as Jurkat, SKBR3, B16F10 and S180, showing promising antitumor properties [17, 18].

Considering these previous findings on BthTX-I and the wide variety of cytotoxic effects presented by snake toxins, additional studies using different tumor cell lines are necessary in order to increase the knowledge of the antitumor and biotechnological potential of this *B. jararacussu* myotoxin. Thereby, this study aimed to evaluate the *in vitro* effects of BthTX-I on human (HL-60 and HepG2) and murine (PC-12 and B16F10) tumor cell lines by assessing its induced cytotoxicity, cell cycle alterations and death mechanisms.

## Methods

### Materials

#### Toxin

BthTX-I was isolated from *Bothrops jararacussu* venom according to the methodology described by Cintra *et al.* [19]. The lyophilized protein was stored at −20 °C and solubilized in phosphate buffered saline (PBS) immediately before its use in the tests.

#### Cell lines

HL-60 (CCL-240, promyelocytic leukemia), HepG2 (HB-8065, human hepatocellular carcinoma), PC-12 (CRL-1721, murine pheochromocytoma) and B16F10 (CRL-6475, murine melanoma) tumor cell lines were obtained from ATCC (American Type Culture Collection, USA).

### Methods

#### Cell culture

Cells were grown in monolayer in 25 cm^2^ flasks. HL-60 cells were cultured in 5 mL of RPMI culture medium (Gibco 31800–022, USA) supplemented with 10 % fetal bovine serum (FBS, Gibco 12657, USA) and 1 % antibiotic (streptomycin and penicillin, P4333 Sigma, USA). PC-12 cells were cultured in 5 mL of RPMI supplemented with 15 % fetal equine serum (Gibco 26050–088, New Zealand), 5 % FBS and 1 % antibiotic. B16F10 and HepG2 cells were grown in DMEM culture medium (Gibco 31600–034, USA) also supplemented with 10 % FBS and 1 % antibiotic. The vials containing the cells were incubated at 37 °C in a humidified incubator containing 5 % CO_2_, until reaching a state of confluence (~5 × 10^6^ cells) when they require subculture. The tests with BthTX-I were performed with cells between the 3rd and 6th day of subculture. Cell viability tests using the Trypan blue dye were performed before any experimentation to ensure the accuracy of the results.

#### Cytotoxic assays using MTT

For the cytotoxicity assay, tumor cells (HL-60, PC-12, HepG2 or B16F10) were seeded into 96-well plates, followed by incubation for 24 h at 37 °C in a humidified incubator containing 5 % CO_2_. After this period, the cells were treated with 50 μL of PBS (negative control) or 50 μL of BthTX-I samples at different concentrations (5; 10; 25; 50 or 100 μg/mL). Experimental positive control received 50 μL of a cisplatin solution at 1 mg/mL (Incel, Darrow®), which is an antineoplastic agent that binds to DNA, inducing structural changes and, consequently, apoptosis. After treatment, the wells received 20 μL of MTT [3-(4,5-dimethylthiazol-2-yl)-2,5-diphenyltetrazolium bromide] (Sigma M2128, USA) (500 μg/mL, final concentration) and plates were incubated for 3 h at 37 °C and 5 % CO_2_. Then, plates were centrifuged at 900 *g* for 5 min and then inverted to discard the supernatant, followed by addition of 100 μL of DMSO (Sigma D2650, USA) to each well. The plates were kept under stirring until complete dissolution of crystals (~20 min) and then the absorbance at 570 nm was determined in a Powerwave XS2 microreader (Biotek) [20].

#### Cell cycle analysis

Tumor cells (HL-60, PC-12, HepG2 or B16F10) were plated into 24-well plates (1 × 10^6^ cells/well), followed by incubation for 24 h at 37 °C in a humidified incubator containing 5 % CO_2_. After this period, the cells were treated with 50 μL of PBS (negative control) or 50 μL of BthTX-I samples at different concentrations (5; 10; 25; 50 or 100 μg/mL). After 24 h of treatment, cells were transferred to flow cytometry tubes and centrifuged at 900 *g* for 5 min, supernatant was discarded and pellet resuspended in 1 mL of PBS. Then, cells were centrifuged again, the supernatant was discarded, the pellet resuspended in 100 μL of cold PBS and 2 mL of cold 70 % ethanol and the samples were stored at −20 °C for 24 h. At the end of this period, the tubes were centrifuged, the supernatant discarded and the pellet was resuspended in 300 μL of cold PBS with 10 μL of RNase (10 mg/mL) and incubated for 30 min at 37 °C. Immediately after the incubation, samples were placed in an ice bath with 164 μL of HFS (hypotonic fluorescence solution, a reagent that measures the amount of DNA in the cell and defines its cell cycle position) and incubated on ice for 1 h, followed by analysis of 10,000 events in a FACSCanto flow cytometer (Becton Dickinson, USA). The files generated by the equipment were read in the FlowJo 7.6.1 software for the analysis of cell division phases: G0/G1, S and G2/M. The results were expressed as the percentage of cells in each phase of the cycle [6].

#### Analysis of apoptosis/necrosis by flow cytometry

The assessment of the apoptotic and necrotic effects induced by BthTX-I on different tumor cell lines (HL-60, PC-12, HepG2 or B16F10) was determined by flow cytometry, using a kit containing annexin V FITC and propidium iodide (PI) [21]. The population of necrotic cells is marked with PI (PI+), while the cells in apoptosis are marked with annexin V FITC (AV+) and the population of both necrotic and apoptotic cells is marked as PI+/AV+.

Tumor cells were plated into 24-well plates (5 × 10^5^ cells/well), followed by incubation for 24 h at 37 °C in a humidified incubator containing 5 % CO_2_. After this period, the cells were treated with 50 μL of PBS (negative control) or 50 μL of BthTX-I samples at different concentrations (5; 10; 25; 50 or 100 μg/mL). Experimental positive control received 50 μL of a cisplatin solution at 1 mg/mL. After 24 h of treatment, cells were transferred to flow cytometry tubes, and centrifuged at 900 *g* for 5 min, supernatant was discarded and pellet resuspended in 1 mL of PBS. The same process was repeated, this time with addition of 300 μL of 1X annexin V to the resuspended pellet and 5 μL of FITC-annexin V to each tube. The samples were incubated for 15 min in ice, adding 1 μL of propidium iodide (PI) immediately after incubation, followed by analysis of 10,000 events in a FACSCanto flow cytometer (Becton Dickinson, USA) using Diva software.

#### Statistical analysis

Results were analyzed by the software Graph Pad Prism 5 using the method one-way ANOVA and Tukey’s post-test, considering values of *p* < 0.05 as significant. All treatments were compared to the negative control (PBS).

## Results

### Evaluation of the cytotoxicity induced by BthTX-I

**Fig. 1:**
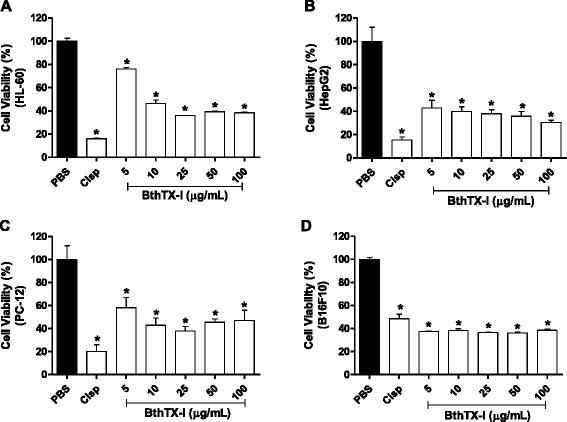
Cell viability of the tumor cell lines HL-60 (**a**), HepG2 (**b**), PC-12 (**c**) and B16F10 (**d**) after treatment with BthTX-I. The cytotoxicity was evaluated by the MTT method 24 h after treatment of tumor cells with BthTX-I (5–100 μg/mL). Results expressed as mean ± SD of three independent experiments (*n =* 3). Statistically significant differences (*p* < 0.05) were labeled with * (in comparison to PBS). PBS (negative control); Cisp (cisplatin, positive control)

The antitumor activity of BthTX-I was assessed by treating tumor cell lines with different concentrations of the myotoxin. For HL-60, it was observed that the cell viability after treatment with BthTX-I was of approximately 80 % for the lowest concentration evaluated (5 μg/mL), while at the concentrations of 10, 25, 50 and 100 μg/mL, cell viabilities of 46; 36; 40 and 38 %, respectively, were observed (Fig. 1 – a). For HepG2, BthTX-I concentrations from 5 to 25 μg/mL led to cell viabilities of approximately 40 %, whereas at the concentrations of 50 and 100 μg/mL, the values of viability observed were of 35 % and 30 %, respectively (Fig. 1 – b). For PC-12, cell viability ranged from 40 to 60 %, with lowest values observed after treatment with BthTX-I at the concentration of 25 μg/mL (Fig. 1 – c). B16F10 cells showed around 40 % cell viability at all concentrations tested (Fig. 1 – d).

### Cell cycle kinetics analysis

**Fig. 2:**
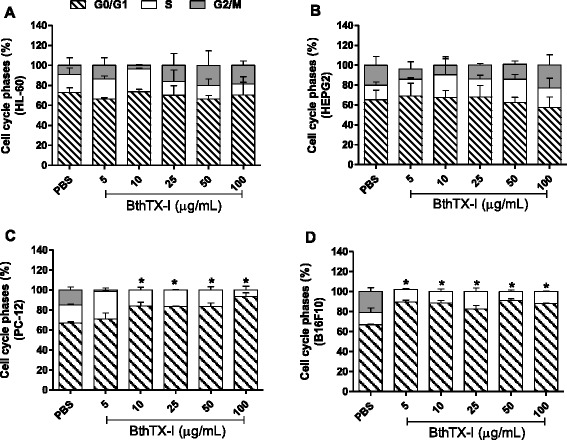
Assessment of cell cycle progression of the tumor cell lines HL-60 (**a**), HepG2 (**b**), PC-12 (**c**) and B16F10 (**d**) treated with BthTX-I using flow cytometry. Tumor cells were treated with BthTX-I at different concentrations (5 to 100 μg/mL). Cells treated with PBS were used as negative control (PBS). The cells were fixed with ethanol and stained with propidium iodide present in the HFS solution. G0/G1: resting/preparation for synthesis; S: DNA synthesis; G2/M: preparation for mitosis. Results expressed as mean ± SD of three independent experiments (*n =* 3). Statistically significant differences (*p* < 0.05) are labeled with * (in comparison with G0/G1 phase of PBS)

The cell cycle kinetics analysis was performed in order to detect the role of BthTX-I in the cell division process of tumor cells. HL-60 and HepG2 cells treated with different concentrations of the toxin showed distribution in all three phases of the cell cycle (G0/G1, S, G2/M), with no significant differences (*p* < 0.05) in comparison with the negative control (PBS). Around 70 % of the HL-60 cells remained in G0/G1, 15 % in S and 15 % in G2/M phases (Fig. 2 – a), while 70 % of HepG2 cells remained in G0/G1, 20 % in S and 10 % in G2/M phases (Fig. 2 – b).

PC-12 and B16F10 cells treated with different concentrations of BthTX-I showed delayed cell cycles, with an increased number of cells in the G0/G1 phase, followed by a reduction in the percentage of cells in the S phase and an absence of cells in the G2/M phase. The increase in the percentage of PC-12 cells in the G0/G1 phase, compared with the negative control, was of ~16 % for the treatments with 10; 25 and 50 μg/mL of BtTX-I, and of 26 % with the toxin at 100 μg/mL (Fig. 2 – c). This increase for B16F10 cells was of approximately 24 % after treatment with BthTX-I at all the concentrations evaluated (Fig. 2 – d).

### Analysis of the apoptotic/necrotic effects of BthTX-I

**Fig. 3:**
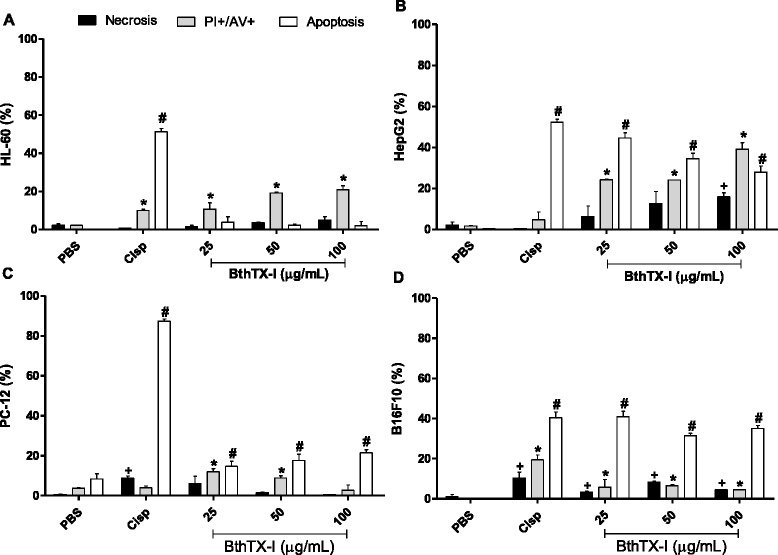
Assessment of apoptotic/necrotic effects of BthTX-I on the tumor cell lines HL-60 (**a**), HepG2 (**b**), PC-12 (**c**) and B16F10 (**d**) by flow cytometry. Tumor cells were treated with BthTX-I at different concentrations (25, 50 or 100 μg/mL). Cisplatin (Cisp, positive control) was used as a reference for the induction of apoptosis and cells treated only with PBS were used as negative control. The population of necrotic cells was labeled with propidium iodide (PI+), apoptosis was labeled with FITC-annexin V (AV+) and population of necrotic and apoptotic cells was labeled with PI+/AV+. Results expressed as mean ± SD of three independent experiments (*n =* 3). Statistically significant differences (*p* < 0.05) were labeled with * (values of PI+/AV+ compared with PBS); # (values of AV+ compared with PBS) or + (values of PI+ compared with PBS)

All concentrations of BthTX-I induced apoptosis/necrosis (PI+/AV+) in leukemic cells (HL-60), with the highest percentage of PI+/AV+ cells (~20 %) observed at the concentrations of 50 and 100 μg/mL. There was also a small percentage of cells that were only in necrotic process (1-5 %) at all the concentrations evaluated (Fig. 3 – a).

The treatment of HepG2 cells with BthTX-I at the concentrations of 25 and 50 μg/mL induced apoptosis/necrosis (PI+/AV+) in 24 % of the cell population, and apoptosis (AV+) in 44 and 34 % of cells, respectively. At the concentration of 100 μg/mL, 16 % of cells were in the process of necrosis (PI+), 40 % in apoptosis/necrosis (PI+/AV+) and 28 % in apoptosis (AV+) (Fig. 3 – b).

BthTX-I at the concentrations of 25 and 50 μg/mL induced about 10 % of PC-12 cell death by apoptosis/necrosis and 15 % by apoptosis. The concentration of 100 μg/mL promoted apoptosis in approximately 20 % of cells (Fig. 3 – c).

The treatment of B16F10 cells with different concentrations of BthTX-I (25, 50 and 100 μg/mL) promoted a high percentage of cell death by apoptosis (AV+) (30 to 40 %), with lower percentages of cells exclusively in the processes of necrosis (3, 8 and 4 %, respectively) or apoptosis (~5 %) (Fig. 3 – d).

## Discussion

Many toxins isolated from snake venoms have been used as tools for understanding different pathophysiological events, since these proteins present several biological effects such as antiparasitic, antimicrobial and antitumor activities [22–24]. In that context, snake venom PLA_2_s of both acidic and basic character, and also synthetic peptides derived from homologous PLA_2_s, have been extensively studied due to their wide variety of pharmacological effects, including their antitumor and antiangiogenic properties [25–27].

In recent years, PLA_2_s have been investigated as tools for better understanding the mechanisms related to cancer. This class of toxins seems to act directly on the metabolism of phospholipid membranes, promoting changes in the lipid biosynthesis and deregulation of lipogenesis in different cell lines, including tumorous ones [28].

In the present study, BthTX-I induced significant cytotoxicity in all tested tumor cell lines. In general, all concentrations of the myotoxin inhibited the viability of HL-60, HepG2, PC-12 and B16F10 cells in 50 to 60 %. These results are consistent with many others which have shown that these toxins have the ability to induce death of various tumor and non-tumor cell lines [11, 29, 30].

Cisplatin (Incel, Darrow®) was used in the present study as a positive control of cytotoxicity, being able to promote death of all tumor cell lines evaluated, with lower effects on B16F10 cells. It is an inorganic platinum compound that binds to DNA and provokes structural changes and inhibition of transcription and replication, thus inducing apoptosis. Considering its cytotoxic effects, cisplatin is widely used as an antineoplastic agent in the treatment of several types of cancer [31].

Gebrim *et al.* [18] evaluated the *in vitro* antitumor activity of BthTX-I using tumor cell lines such as Jurkat, SKBR3 and B16F10, and showed that the native protein presented 75 to 90 % cytotoxicity on these tumor cell lines. Regarding *in vivo* experiments, injection of BthTX-I modified with p-bromophenacyl bromide (BPB, an agent that inhibits PLA_2_s by covalently binding to their active site) in mice transplanted with S180 tumor cells reduced 30 % of the tumor size on the 14th day and 76 % on the 60th day, when compared to the untreated control group. These findings are in accordance with other studies that suggested that the antitumor properties of PLA_2_s are independent of their catalytic activity [32, 33].

De Moura *et al.* [11] evaluated the cytotoxic effect of three PLA_2_s isolated from *B. mattogrossensis* venom, BmatTX-I (Lys49), BmatTX-II (Lys49) and BmatTX-III (Asp49) on Jurkat and SKBR-3 tumor cells. These authors observed that the Lys49 myotoxins were more cytotoxic than the Asp49 PLA_2_ to both tumor cells. Similar to that observed for BthTX-I, the toxins BmatTX-I and BmatTX-II at a concentration of 100 μg/mL inhibited the cell viability of Jurkat cells in approximately 50 %. Equivalent effects were observed when Jurkat cells were treated with 100 μg/mL of two acidic Asp49 PLA_2_s, BmooTX-I from *B. moojeni* venom and MTX-I from *B. brazili* venom [34, 35]. BthA-I-PLA_2_, an acidic PLA_2_ from *B. jararacussu* venom, also showed potential antitumor effects on Jurkat, SKBR-3 and Ehrlich ascites tumor (EAT) cells, with the enzyme at 100 μg/mL promoting death of 50 to 70 % of the tumor cells [36].

Studies suggest that several biological effects of the myotoxins are related to the interaction of their C-terminal region with cell membranes, which explains the synthesis of peptides derived from this region described in different studies [18, 35, 37]. The C-terminal region is usually described as capable of disrupting the hydrophilic matrix of membranes, opening pores and allowing the entrance of the toxins into the intracellular medium [38].

Investigations involving the mechanisms of action of snake venom toxins on tumor cells are still scarce in the literature. However, some suggestions of mechanisms have been proposed for components such as L-amino acid oxidases (LAAOs), which have been described as potent cytotoxic agents. Several studies attributed the cytotoxic effects of LAAOs mainly to the release of hydrogen peroxide to the medium, resulting in oxidative stress and consequently in cell death [7, 39].

The effect of cytotoxic agents can be evaluated by cell cycle analyses. The cell cycle is a series of continuous and repetitive processes that occur during a normal cell division [40]. The cycle is divided into interphase and mitosis. The interphase is further subdivided into the phases G0, G1, S and G2, in which the DNA duplication and preparation of cells for the next stage occurs. The mitosis is characterized by the cell division process itself [40, 41].

Flow cytometry experiments were performed for the cell cycle analyses, with the purpose of evaluating the effects of BthTX-I in the process of cell division. Nuclear DNA of cells was stained by a specific fluorochrome (PI), allowing its quantification and reflecting the position of cells in the cell cycle. BthTX-I was not able to induce any significant change in the cell cycle of HL-60 and HepG2 tumor cells. Conversely, the toxin caused a delay in the G0/G1 phase of the cell cycle of PC-12 and B16F10 tumor cells. The fact that the myotoxin promoted delay in the cell cycle indicates that it disrupts the mitotic progression and hence a smaller number of cells are doubled, which would implicate an important antitumor mechanism.

Although few studies have been conducted with snake venom toxins regarding their effects on the cell cycle progression, some other snake toxins were also described as able to promote delay of the cell cycle in the G0/G1 phase [6, 42]. Checkpoint proteins (CKI’s, CDK’s and CHK) are probably preventing these cells from leaving the G0/G1 phase. The CKI’s are able to suspend the cell cycle at any sight of DNA damage, directing the cells to repair systems. The cell cycle only continues when the damaged DNA is restored, and in case the repair systems are unable to correct the damage, cell death processes are induced [43].

Exposure to toxic agents can trigger cell death by processes of apoptosis or necrosis, and both processes occur depending on the intensity and duration of exposure. Necrosis is an accidental form of cell death in which a failure in the cell homeostasis occurs after the cell is damaged, leading to an inflammatory process [44–46]. Apoptosis is a controlled form of cell death, defined by a number of biochemical and morphological characteristics, such as the exposure of phosphatidylserine to the outside of the plasma membrane, the cell nucleus condensation and the cleavage of chromatin (DNA) in oligonucleosomal fragments [47].

The apoptosis-inducing ability of BthTX-I was assessed by flow cytometry using annexin V conjugated to fluorescein, which acts as a marker for the phosphatidylserine exposed on cells undergoing apoptosis stage. The necrotic effect was analyzed by staining with propidium iodide (PI), which marks the nucleic acid of dead cells.

Our results showed that different BthTX-I concentrations induced tumor cell death by processes of apoptosis and/or necrosis. The myotoxin mainly induced apoptosis (AV+) in PC-12 and B16F10 cells at all concentrations tested (25, 50 or 100 μg/mL), which suggests this process as the predominant cell death mechanism induced by BthTX-I on these murine tumor cells. Regarding human tumor cell lines (HL-60 and HepG2), apparently distinct death mechanisms could be observed: the percentage of HepG2 cells in apoptosis (AV+) was practically equal to those in apoptosis/necrosis (PI+/AV+), while for HL-60 cells, the major percentage of cells was marked as PI+/AV+, indicating death mechanisms related to both apoptosis and necrosis.

These findings corroborate the data obtained for other enzymes isolated from snake venoms, which promoted death in various cell lines by different mechanisms, such as apoptosis and necrosis [48, 49]. Several factors may determine whether cells go into apoptosis or necrosis, such as the toxin concentration, the ability of cells to manage local membrane perturbations and the metabolic status of cells [50]. Mora *et al.* [9] evaluated the induction of apoptosis and necrosis by a Lys49 PLA_2_ homologue from *Bothrops asper* snake venom in a lymphoblastoid cell line, and concluded that the enzyme was able to induce significantly different effects depending on the concentration of toxin used.

## Conclusion

BthTX-I was shown to be cytotoxic for both human (HepG2 and HL-60) and murine (PC-12 and B16F10) tumor cell lines by inducing cell death mechanisms of apoptosis and/or necrosis. Additionally, BthTX-I was able to promote delay in the G0/G1 phase of the cell cycle of murine tumor cells. The results revealed important functional characteristics of this myotoxin isolated from *B. jararacussu* venom, demonstrating a remarkable antitumor potential still little explored. Studies on the mechanisms of action of BthTX-I are very important so that it may serve as a model for the development of new therapeutic agents to treat various human diseases such as cancer in the future.
